# Functional response of *Amblyseius eharai* (Acari: Phytoseiidae) on *Tetranychus urticae* (Acari: Tetranychidae)

**DOI:** 10.1371/journal.pone.0260861

**Published:** 2021-12-02

**Authors:** Young-Gyun Park, Joon-Ho Lee, Un Taek Lim

**Affiliations:** 1 Department of Agricultural Biotechnology, Entomology Program, Seoul National University, Seoul, Republic of Korea; 2 Research Institute of Agriculture and Life Sciences, Seoul National University, Seoul, Republic of Korea; 3 Department of Plant Medicals, Andong National University, Andong, Republic of Korea; University of Carthage, TUNISIA

## Abstract

*Amblyseius eharai* is a generalist predatory mite that consumes spider mites, rust mites, thrips, and pollen, with a high adaptability to various plants. To better understand ecological and behavioral aspects of this species, we investigated its functional response to different stages of two-spotted spider mite, *Tetranychus urticae*. Furthermore, we compared its environmental adaptability with that of other referenced phytoseiids using a temperature-dependent model of the intrinsic rate of increase. We were able to calculate the functional response parameters of both sexes of *A*. *eharai* when preying on eggs or larvae of *T*. *urticae* and, for females only, when preying on the deutonymph of *T*. *urticae*. Among the various combinations tested herein, *A*. *eharai* females preying on *T*. *urticae* larvae had the highest attack rate and shortest handling time. For eggs of *T*. *urticae*, *A*. *eharai* showed a lower attack rate; however, its handling time for eggs was significantly shorter compared to other phytoseiids. Using *T*. *urticae* larva as a prey, the attack rate of female *A*. *eharai* was higher and the handling time of both sexes of this species was shorter than those of other phytoseiid mites. *Amblyseius eharai* populations can show maximum performance quickly due to this species’ lower optimal temperature for population growth (28.1°C) compared to other phytoseiid mites. Thus, we provided evidence that this predatory mite has the potential to be a new, effective biological control agent of greenhouse pests such as *T*. *urticae* due to its high predation capacity and low optimal temperature.

## Introduction

Predatory mites are very effective biological control agents and are commonly used to control small-bodied pests such as spider mites, thrips, and whiteflies in agricultural crops [1, 2]. Phytoseiid mites are among the most extensively commercialized and frequently applied natural enemies for augmentative biological control in greenhouses [3–6]. Among them, *Amblyseius swirskii* (Athias-Henriot), *Neoseiulus californicus* (McGregor), and *Phytoseiulus persimilis* (Athias-Henriot) (Acari: Phytoseiidae) are widely commercialized species.

*Amblyseius eharai* (Amitai et Swirski) (Acari: Phytoseiidae) is a native species in East Asia and is a type III generalist predator [2, 3, 7]. It feeds on spider mites, eriophyid mites, thrips, and pollen [2, 3, 8–11] and has a lower optimal temperature for development and oviposition compared to other predatory mites such as *A*. *swirskii*, *N*. *californicus*, *N*. *longispinosus* (Evans), and *N*. *womersleyi* (Schicha) [11–15]. Park and Lee [11] reported that *A*. *eharai* did not oviposit at 33.2°C nor were its larvae able to develop at 35.9°C, while in comparison, *A*. *swirskii*, *N*. *californicus*, *N*. *longispinosus*, and *N*. *womersleyi* all showed better development and reproduction than *A*. *eharai* at 36.0°C [12–15]. Despite these ecological adaptations for lower temperature conditions, *A*. *eharai* is potentially a valuable biological control agent since it has a high adaptability to many types of plants such as deciduous trees, conifers, shrubs, herbs, and vine [16]. Especially, this predator would adapt easily to East Asian agricultural land to which it is native. However, there is limited information available on other ecological aspects of *A*. *eharai*, especially on its functional response, which can be important when developing a new species for use as a biological control agent. Knowledge of a species’ type of functional response to a particular pest can help evaluate its potential control efficacy in a particular environment [17]. Type II and III are the most common functional response types in predatory arthropods [18–23], but these patterns can be affected by the physiological status of either the predator or prey species [20, 21]. From the functional response type, it is possible to estimate a predator’s attack rate and handling time. Although promising parameter values for a predator’s functional response do not guarantee its success for use in a biological control program, they can be used to assess a predator’s potential relative to other species in the same predator guild.

The two-spotted spider mite, *Tetranychus urticae* (Koch) (Acari: Tetranychidae), is a worldwide pest that can cause severe damage to many horticultural crops in greenhouses and open fields [24–26]. Spider mites reduce crop photosynthesis by feeding on plant cells and are hard to control due to their high population growth rates on many crops [24, 27, 28] and their rapid development of resistance to pesticides [29, 30]. As an alternative to chemical insecticides, biological control agents including predatory mites, anthocorid bugs, and pathogenic fungi can be applied [31, 32]. For the successful biological control of *T*. *urticae* in farmland, agents used should be well adapted to the area’s environmental and ecological conditions [31, 33].

In this study, we evaluated functional response of *A*. *eharai*, a phytoseiid native to East Asia, on different development stages of *T*. *urticae* to explore the potential of this predator as a biological control agent. Additionally, the attack rate and handling time of *A*. *eharai* on eggs and larvae of *T*. *urticae* were calculated and compared with those of other phytoseiid mites used as biocontrol agents. Finally, to estimate where *A*. *eharai* might function well for biological control, we compared its environmental adaptability with other predatory mites using a temperature-dependent model of intrinsic rate of increase values obtained from the scientific literature.

## Materials and methods

### Laboratory rearing of predator and prey mites

*Tetranychus urticae* was reared in the Laboratory of Insect Ecology, Seoul National University using kidney bean plants as food. Two to three kidney beans were seeded in a pot (85 × 70 mm; diameter × height) and grown for two weeks before being used to rear *T*. *urticae*. Six to seven such potted plants were inoculated with mixed stages of *T*. *urticae* (over 1000 mites) and then placed on a small steel tray (365 × 320 × 50 mm; width × length × height). This small tray was then placed in a bigger steel tray (550 × 455 × 60 mm; width × length × height) that was flooded with water to prevent mite escape. About half of the plants in a small tray were replaced with new ones every 3–4 days. The stems and leaves of plants being removed were cut off and draped over the new plants to transfer their *T*. *urticae* individuals. Rearing was done at 25°C, 60–80% RH, and a photoperiod of 16:8 (L:D) h in an insectary.

*Amblyseius eharai* used in our experiments were from a colony started with mites collected from the overwintering grape buds of vineyards in Hwaseong, Korea (N 37° 10’ 23.5", E 126° 41’ 39.1"). A total 150 *A*. *eharai* females were collected from the buds and kept at 26–8°C, 60–80% RH, and a photoperiod of 16:8 (L:D) h in an incubator (50 × 50 × 50 cm; width × length × height; Hanbaek Scientific Technology, Bucheon, Korea). To provide food for the predatory mites, mixed stages of *T*. *urticae* on an infested kidney bean leaf from the rearing colony were placed in each Petri dish (100 × 42 mm; diameter × height) (SPL life science, Pocheon, Korea) used to rear predators. The Petri dishes with the predatory mites each contained an excised kidney bean leaf disc (70 mm diameter, upside down) (infested with *T*. *urticae*) on water-saturated cotton. Each Petri dish was inoculated with 10 to 15 *A*. *eharai* females. The kidney bean leaves infested with *T*. *urticae* in these dishes were replaced with new ones from the spider mite rearing colony once a week. When leaves with prey were replaced, all detectable *A*. *eharai* stages including eggs were transferred onto the new prey-infested leaves with a fine brush. At least 30 to 40 generations of *A*. *eharai* were produced in this way before the colony was used in this experiment.

### Functional response experiment

To assess the functional response of *A*. *eharai*, the experiments were conducted at 26.6 ± 0.39°C, 72.2 ± 2.51% RH, and a photoperiod of 16:8 (L:D) h using adults of *A*. *eharai* of our rearing colony as described above. To obtain freshly emerged *A*. *eharai* adults, deutonymphs were collected from the rearing colony and isolated in a Petri dish under the same conditions as used for rearing. The immature development of these mites was checked daily, and newly molted adults of both sexes were moved to another Petri dish (held under the same conditions) and allowed to feed, mate, and lay eggs for three days. After three days, adults (now 3-day-old) of each sex were placed individually in Petri dishes with a single kidney bean leaf with no prey mites and starved for 24 h.

Smaller Petri dishes (50 × 15 mm; diameter × height; SPL Life Science, Pocheon, Korea) were used as test arenas for the experiments. In each test Petri dish, one kidney bean leaf disc (35 mm diameter) was placed upside down on water-saturated cotton. Preys (*T*. *urticae*) in various densities were released in these Petri dishes with predators (*A*. *eharai*). The densities of each prey stage and its replication numbers for the functional response experiments are presented in Table 1.

**Table 1:** Prey densities tested for the functional response test of *Amblyseius eharai* (both males and females) when fed various life stages of *Tetranychus urticae* as prey.

Prey stage	Sex of *A*. *eharai*	Prey densities (replication number)
Egg	F (female)	10 (11), 30 (11), 50 (10), 70 (10), 130 (11)
	M (male)	10 (10), 30 (10), 50 (10), 70 (10), 130 (10)
Larva	F	10 (11), 30 (11), 50 (10), 70 (10), 130 (10)
	M	10 (10), 30 (10), 50 (12), 70 (10), 130 (11)
Protonymph	F	10 (11), 20 (10), 30 (11), 50 (10), 80 (10)
	M	10 (10), 20 (11), 30 (11)
Deutonymph	F	10 (10), 20 (11), 30 (10), 40 (11), 70 (10)
	M	10 (11), 20 (10), 30 (10), 40 (10), 70 (10)
Adult female	F	5 (10), 10 (11), 15 (10), 20 (11), 40 (11)
	M	5 (10), 10 (10), 15 (10), 20 (10)

To obtain *T*. *urticae* eggs for experiments, five to ten adult female mites collected from the rearing colony were placed in each intended test dish, where they were allowed to lay eggs for 24 hours. Adult mites were then removed, and the number of eggs counted. The number of eggs per dish (Table 1) was adjusted by removing or adding eggs with a fine brush. To obtain larvae and protonymphs of *T*. *urticae*, 300 to 400 females of *T*. *urticae* were placed in each of two to three Petri dishes (100 × 42 mm; diameter × height), on a kidney bean leaf disc (70 mm diameter, upside down) placed upside down on water-saturated cotton. The *T*. *urticae* females were allowed to lay eggs for 48 hours, and then the females were removed. For three to seven days, daily observations were made to check the developmental stage of the *T*. *urticae* progeny. When mites were larvae or protonymphs, they were moved to the smaller (50 × 15 mm, diameter x height) test Petri dishes for experiments. The higher prey stages (deutonymphs and adult females of *T*. *urticae*) needed for the functional response experiments were collected directly from the rearing colony and moved to test Petri dishes. In the process of gathering prey mites, webbing of mites was excluded.

The starved adult females or males of *A*. *eharai* were individually placed in the test Petri dishes where the prey has been introduced. The *A*. *eharai* adults were allowed to attack prey for 24 hours, and the number of preys consumed were then counted. If the adult *A*. *eharai* died, that replicate was discarded.

### Prey stage preference test (eggs vs. larvae of *T*. *urticae*)

For adult males and females of *A*. *eharai* we compared the numbers of prey consumed for eggs vs. larvae of *T*. *urticae*. This test was run under the same environmental conditions as the functional response experiment. The eggs and larvae of *T*. *urticae* were obtained by the same method as used in functional response experiments. Twenty-five eggs or larvae were transferred to the same kidney bean leaf disc (35 mm diameter, upside down) on water-saturated cotton in a test Petri dish (50 × 15 mm, diameter × height) with a fine brush. Adult females and males of *A*. *eharai* were obtained by the same method as used in functional response experiments, and they were placed individually in the test Petri dishes containing *T*. *urticae* eggs and larvae on a leaf disc. The predatory mites were allowed to feed on eggs and larvae of *T*. *urticae* for 24 hours, and then the numbers of eggs or larvae consumed were determined by counting the survivors. Eleven replications were run for females and ten for males.

### Data analysis

The effects of prey density, life stage (egg vs. larva), and predator sex on the predation rate were analyzed by three-way analysis of variance (ANOVA) using PROC GLM in SAS (SAS Institute, Cary, NC) [34]. The effects of the prey density and predator sex on the predation of protonymphs, deutonymphs, and adult females were all analyzed by two-way ANOVA using PROC GLM in SAS (SAS Institute, Cary, NC) [34]. In the case of adult female predators, data on protonymph and adult female prey were analyzed using one-way ANOVA with PROC GLM in SAS (SAS Institute, Cary, NC) [34] due to lack of the male data in some density treatments. The numbers of consumed *T*. *urticae* eggs and larvae in the prey preference test were compared using a *t*-test with PROC TTEST in SAS (SAS Institute, Cary, NC) [34].

The functional response data were analyzed in two phases as per Juliano [35], using PROC GENMOD in SAS (SAS Institute, Cary, NC) [34]. Logistic regression was performed to assess the form of the functional response. The equation is:

$$\frac{N_{e}}{N_{0}}=\frac{exp(P_{0}+P_{1}N_{0}+P_{2}N_{0}^{2}+P_{3}N_{0}^{3})}{1+exp(P_{0}+P_{1}N_{0}+P_{2}N_{0}^{2}+P_{3}N_{0}^{3})}$$ (1)

where *N*_e_ is the number of prey consumed per predator, the initial number of prey is *N*_0_, and the likelihood of consumption is *N*_e_/*N*_0_. The parameters include *P*_0_, *P*_1_, *P*_2_, and *P*_3_.

Using logistic regression, maximum likelihood estimates were obtained for parameters *P*_0_ to *P*_3_. To determine the functional response type, the logistic model parameter was estimated by the log-likelihood test. If *P*_1_ < 0, the functional response is type II. If *P*_1_ > 0 and *P*_2_ < 0, the functional response is type III [35]. In this study, all significant (*P* < 0.05) *P*_1_ values of *A*. *eharai* on different prey stages were negative. Thus, for type II functional responses, the random predator equation [36] was used to estimate functional response parameters such as an attack rate (*α*) and handling time (*T*_h_). The equation is:

$$N_{e}=N_{0}[1-\text{exp}\left(\alpha T_{h}N_{e}-\alpha T\right)]$$ (2)

where *N*_e_ is the number of preys consumed per predator during the test time *T* (24 h), *N*_0_ is the initial prey number. We compared the attack rate and handling time of *A*. *eharai* among the treatments at a 95% confidence interval.

## Results

### Predation rates of *Amblyseius eharai* on *Tetranychus urticae* life stages

Predation rates of *A*. *eharai* on *T*. *urticae* eggs and larvae were affected by prey density, prey stage, *A*. *eharai* sex, the prey density × stage interaction, and the prey density × *A*. *eharai* sex interaction (Table 2). However, predation rates were not significantly affected by the prey density × stage × *A*. *eharai* sex interaction (Table 2). Predation rates for *A*. *eharai* sexes on different densities of *T*. *urticae* larvae or eggs showed that as the initial prey density increased, the predation rate of *A*. *eharai* also increased (Table 3). The predation rate by female *A*. *eharai* was generally higher on larvae than on eggs of *T*. *urticae* (Table 3).

**Table 2:** ANOVA results of predation rate of *Amblyseius eharai* on different stages of *Tetranychus urticae*.

Prey	ANOVA	Source	*df*	*F*	*P*
Egg vs. Larva	3-way	Density	4	165.12	< 0.001
		Prey	1	26.24	< 0.001
		Sex	1	309.62	< 0.001
		Density × Prey	4	3.33	0.012
		Density × Sex	4	36.72	< 0.001
		Prey × Sex	1	15.61	< 0.001
		Density × Prey × Sex	4	0.92	0.455
		Model	19	61.10	< 0.001
		Error	188		
Protonymph	2-way	Density	2	57.26	< 0.001
		Sex	1	196.83	< 0.001
		Density × Sex	2	14.38	< 0.001
		Model	5	68.23	< 0.001
		Error	58		
	1-way (Female)	Model	4	28.85	< 0.001
		Error	47		
Deutonymph	2-way	Density	4	42.06	< 0.001
		Sex	1	23.82	< 0.001
		Density × Sex	4	235.26	< 0.001
		Model	9	11.58	< 0.001
		Error	93		
Adult female	2-way	Density	3	2.85	0.043
		Sex	1	117.96	< 0.001
		Density × Sex	3	3.98	0.011
		Model	7	19.82	< 0.001
		Error	74		
	1-way (Female)	Model	4	4.62	0.003
		Error	48		

**Table 3:** Comparison of predation rate (as number of prey consumed, mean ± SE) of *Amblyseius eharai* adults (F: Female or M: Male) on eggs and larvae of *Tetranychus urticae*.

Prey density	Prey stage			
	Egg		Larva	
	F	M	F	M
10	6.9 ± 0.89d^†^ A^‡^	5.3 ± 0.67c A	9.4 ± 0.20d A	6.1 ± 0.80c A
30	18.8 ± 2.37cd AB	13.2 ± 1.85bc AB	26.7 ± 0.30c A	12.4 ± 0.79bc B
50	26.9 ± 2.29c AB	13.3 ± 1.95bc BC	39.6 ± 1.43c A	13.0 ± 0.91bc C
70	47.7 ± 2.28b A	20.6 ± 2.05b B	58.1 ± 2.74b A	21.2 ± 2.10ab B
130	63.1 ± 4.78a B	27.0 ± 5.27a C	85.5 ± 6.47a A	33.9 ± 4.04a C

^†^Means followed by the same letter within each column are not significantly different at α = 0.05, Tukey’s studentized range test.

^‡^Means followed by the same letter within each row are not significantly different at α = 0.05, Tukey’s studentized range test.

In the treatment of protonymph, deutonymph, and adult females of *T*. *urticae* to *A*. *eharai*, the predation rate was affected by prey density, sex of *A*. *eharai*, and their interaction (Table 2). In general, female predatory mites consumed more *T*. *urticae* than males (Table 4). Female *A*. *eharai* consumed significantly more prey at higher prey densities than at lower densities. However, such trend was not found in male *A*. *eharai* that rarely preyed on *T*. *urticae* adult female.

**Table 4:** Predation rate (as number of preys consumed, mean ± SE) of *Amblyseius eharai* on *Tetranychus urticae* in the protonymph, deutonymph, and female adult life stages.

Prey		No. of Preys Consumed (for Both Predator Sexes)	
Stage	Density	Female	Male
Protonymph	10	8.9 ± 0.46d^†^ A^‡^	4.2 ± 0.44b B
	20	15.1 ± 0.60cd A	5.5 ± 0.77ab B
	30	21.7 ± 1.32bc A	8.5 ± 0.79a B
	50	28.2 ± 3.32ab	-
	80	32.8 ± 1.94a	-
Deutonymph	10	6.3 ± 0.80b A	2.7 ± 0.30a A
	20	7.7 ± 0.90b A	3.8 ± 0.33a B
	30	14.1 ± 0.94a A	3.6 ± 0.34a B
	40	16.1 ± 1.20a A	4.9 ± 0.64a B
	70	16.8 ± 1.42a A	5.3 ± 0.67a B
Adult female	5	2.7 ± 0.30b A	0.5 ± 0.17a A
	10	4.7 ± 0.57ab A	0.3 ± 0.15a B
	15	6.2 ± 0.93ab A	0.2 ± 0.13a B
	20	4.4 ± 0.94ab A	0.2 ± 0.13a B
	40	7.5 ± 1.18a	-

^†^Means followed by the same lower-case letter within a column in each prey stage are not significantly different at α = 0.05, Tukey’s studentized range test.

^‡^Means followed by the same capital letter within a row in each prey stage and density are not significantly different at α = 0.05, Tukey’s studentized range test.

### Prey preference of *A*. *eharai* between *T*. *urticae* eggs and larvae

**Fig 1:**
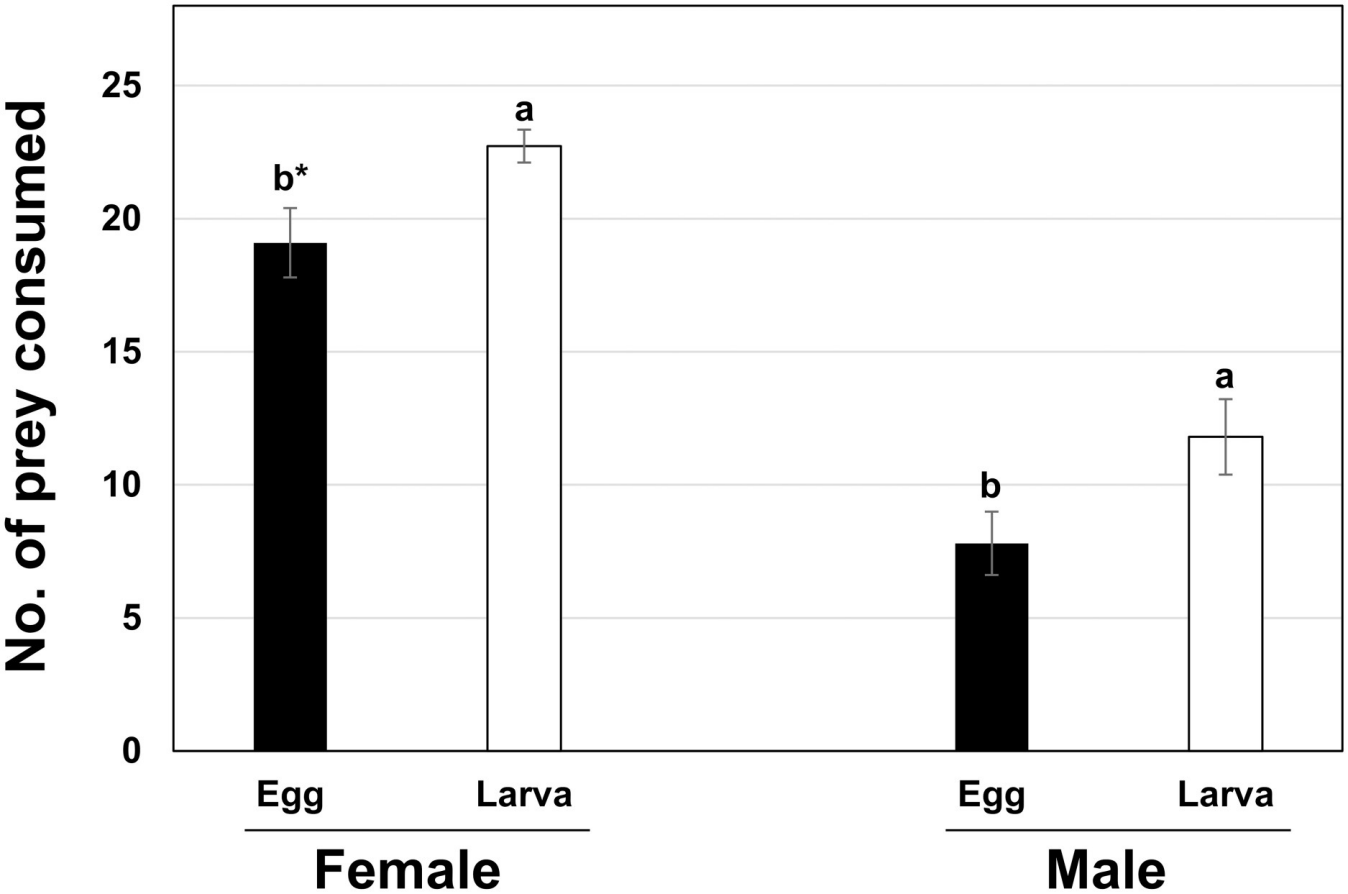
Comparison of predation rate of *A*. *eharai* on egg and larva of *T*. *urticae* at the same petri dish. *Means followed by the same letter in each sex are not significantly different at α = 0.05, *t*-test.

The results of prey preference of *A*. *eharai* are presented in Fig 1. Both sexes of *A*. *eharai* consumed more *T*. *urticae* larvae than eggs (Female, *T*_14.30_ = 2.52, *P* = 0.024; Male, *T*_18_ = 2.16, *P* = 0.045). Female *A*. *eharai* consumed 22.7 larvae and 19.1 eggs of *T*. *urticae* during 24 hours. Male *A*. *eharai* consumed 11.8 larvae and 7.8 eggs of *T*. *urticae* during 24 hours.

### Functional response

**Fig 2:**
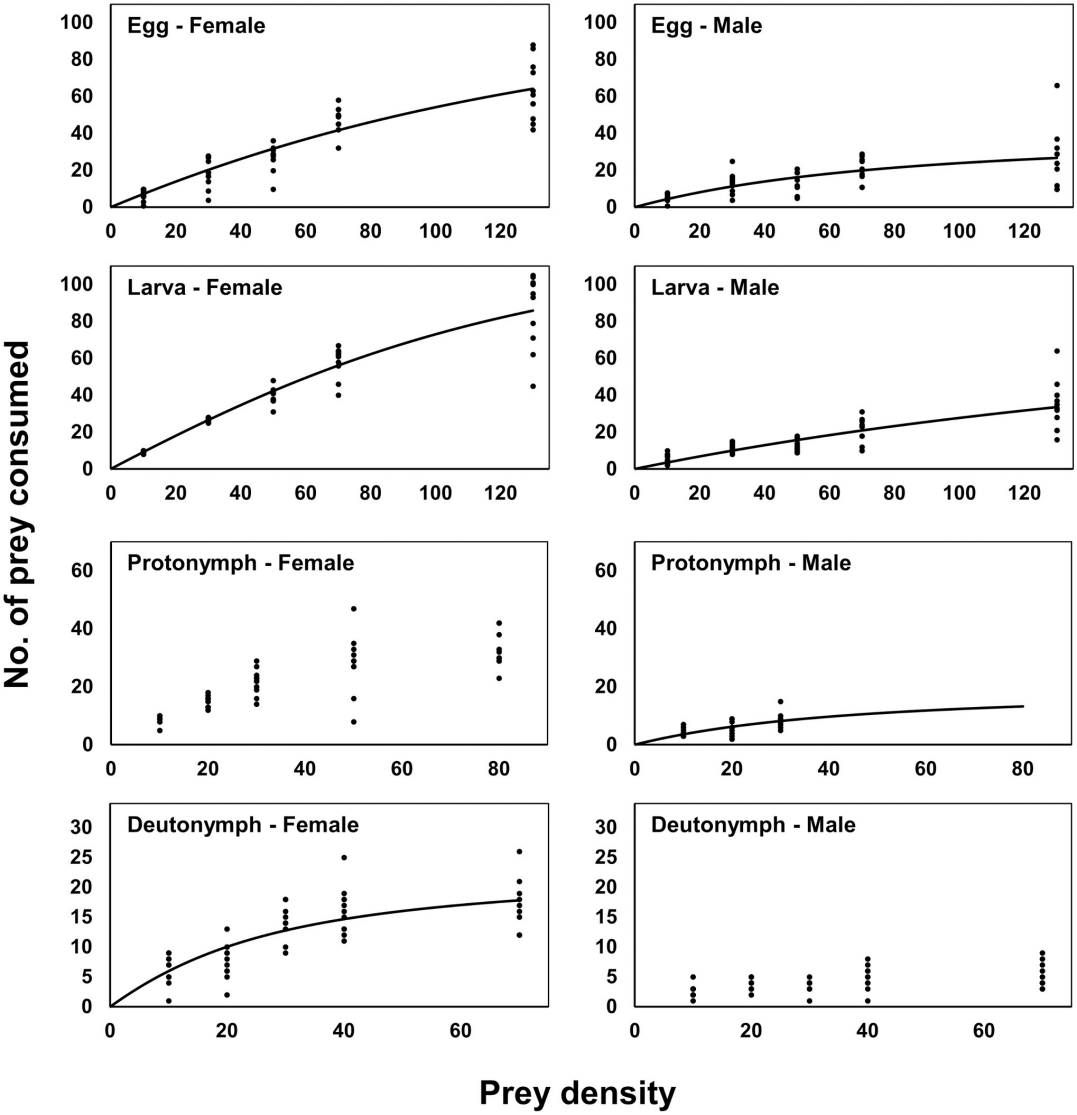
Functional response of *Amblyseius eharai* to eggs, larvae, protonymphs, and deutonymphs of *Tetranychus urticae* on kidney bean leaf discs (35 mm diameter) over 24 h.

In the treatment that showed significance of *P*_1_ value (Egg for females, *P* < 0.001; Egg for males, *P* = 0.003; Larva for females, *P* = 0.001; Larva for males, *P* < 0.001; Deutonymph for females, *P* = 0.015; Adult for males, *P* = 0.001), the *P*_1_ values for the relations between prey presented and prey consumed all were negative, indicating a type II functional response (Table 5, Fig 2). The attack rate was the highest in larval mite treatment fed on by female *A*. *eharai* (0.1087), and while it was lowest, also for larval mites as prey but fed on by male *A*. *eharai* (0.0186). Handling time was shortest for larval mites fed on by female *A*. *eharai* (0.1642) and longest for deutonymphs fed on by female *A*. *eharai* (1.0232). For protonymphs fed on by female *A*. *eharai* the relationship was not statistically significant (*P* = 0.108). For deutonymphs, the attack rate and handling time of female *A*. *eharai* were 0.0224 and 1.2105. Finally, the *P*_1_ values for male predators attacking deutonymphs and female predators attacking adult mites were both not significant (*P* = 0.116 and *P* = 0.181, respectively). Moreover, even though the *P*_1_ value for male predators attacking adult prey was significant (*P* = 0.014), this treatment was excluded in the comparison of parameters and model fitting because of its higher standard error of parameters.

**Table 5:** Estimates of parameters (mean ± SE) for *Amblyseius eharai* preying on different stages of *Tetranychus urticae* for 24 hours: Attack rate, handling time, and *P*_1_ value from the random predation equation and logistic regression.

Prey stage	Sex of predatory mite	Attack rate	Handling time	Maximum likelihood estimate			*r* ^2^
		(*α* ± S.E.) (95% C.I.)	(*T*_h_ ± S.E.) (95% C.I.)	*P*_1_ (± S.E.)	χ^2^	*P*	
Egg	F	0.0548 ± 0.0103ab* (0.0341–0.0756)	0.1808 ± 0.0473bc (0.0858–0.2758)	-0.092 ± 0.0219	17.50	< 0.001	0.94 (*F*_2,51_ = 413.21, *P* < 0.001)
	M	0.0268 ± 0.0089bc (0.0090–0.0447)	0.5779 ± 0.1704ab (0.2353–0.9206)	-0.065 ± 0.0216	9.04	0.003	0.80 (*F*_2,48_ = 98.30, *P* < 0.001)
Larva	F	0.1087 ± 0.0231a (0.0624–0.1550)	0.1642 ± 0.0310c (0.1019–0.2264)	-0.124 ± 0.0387	10.23	0.001	0.97 (*F*_2,50_ = 690.56, *P* < 0.001)
	M	0.0186 ± 0.0037c (0.0111–0.0260)	0.2343 ± 0.1227bc (-0.0120–0.4807)	-0.105 ± 0.0216	23.73	< 0.001	0.89 (*F*_2,51_ = 202.10, *P* < 0.001)
Protonymph	F	-		-0.085 ± 0.0530	2.59	0.108	-
	M	-					
Deutonymph	F	0.0506 ± 0.0116ab (0.0274–0.0739)	1.0232 ± 0.1377a (0.7466–1.2999)	-0.157 ± 0.0640	5.98	0.015	0.93 (*F*_2,50_ = 318.33, *P* < 0.001)
	M	-		-0.127 ± 0.0811	2.47	0.116	-
Adult female	F	-		0.228 ± 0.1706	1.79	0.181	-
	M^†^	0.1678 ± 1.5131 (-2.8953–3.2309)	185.9 ± 131.9 (-81.0–452.9)	-0.585 ± 0.1690	12.00	0.001	0.20 (*F*_2,50_ = 4.81, *P* = 0.014)

*Means followed by the same letter within a column are not significantly different at approximate 95% confidence interval.

^†^Excluded in the comparison.

## Discussion

In this study, we explored the predation traits of adult *A*. *eharai* on the different developmental stages of *T*. *urticae*, and, as far as we know, this is the first study on the functional response of *A*. *eharai*. *Amblyseius eharai* showed a type II functional response against eggs, larvae, and deutonymphs of *T*. *urticae*. Although the type of functional response can vary among prey species or the predator’s physiological condition, type II is a common response in predatory mites [18–22, 37].

**Fig 3:**
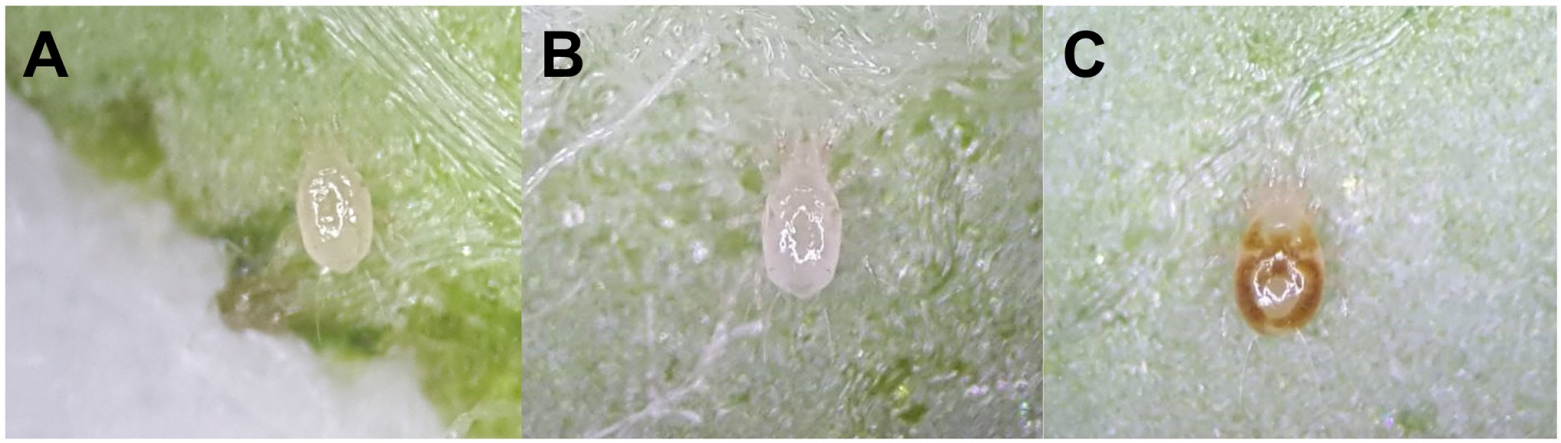
Comparison of pictures of female *A*. *eharai* according to prey consumed conditions. (A) Starved female for 24 hours. (B) Female that consumed *T*. *urticae* eggs for 24 hours. (C) Female that consumed *T*. *urticae* larvae for 24 hours.

Also, predatory mites can be divided into four dietary groups, with most generalist predators belonging to types III or IV [2, 3], preying on various small-sized pests but also ingesting pollen. Which prey and pollen are most suitable for particular species of type III or IV phytoseiid mites vary depending on predator species [2, 3]. In our study, both sexes of *A*. *eharai* preferred *T*. *urticae* larvae over eggs. Blackwood et al. [38] reported that type III generalists either preferred *T*. *urticae* larvae over eggs, or showed no preference between the two. *Amblyseius eharai* is a type III generalist [2, 3], with a preference for *T*. *urticae* larvae. Interestingly, during our predation tests, we noticed variation in body shape of the predatory mites depending on prey life stage they had consumed (Fig 3). While starved females or one that had consumed eggs seemed to have a similar appearance, with a translucent body color and narrower body width (Fig 3A and 3B), females that had consumed larvae had a brownish color for their digestive tack and broader body (Fig 3C). Gut emptying could be used as a physiological indicator of phytoseiids [39, 40]. In Fig 3B, its gut is empty despite the food consumption, thus the predator probably has poor nutritional condition. Based on our results, we conclude that *T*. *urticae* eggs are a poor quality food for *A*. *eharai* adults.

Webbing of spider mites is known to serve as a shield for them, and it can mediate the anti-predator behavior [41]. In this study, any webbing was discarded from the experimental arena, thus having potential of affecting the outcome of functional response analysis in the study.

In our study, *A*. *eharai* females had higher predation rates than males, for all *T*. *urticae* life stages. Moreover, *A*. *eharai* males usually failed to consume *T*. *urticae* adults. Like other phytoseiid mites, the body size of female *A*. *eharai* (length of dorsal shield: 0.352 mm) is greater than that of males (length of dorsal shield: 0.266 mm) [7]. Higher predation by females than by males and the male’s failure to prey on *T*. *urticae* adults may be a simple function of body size.

The attack rates and handling times of male and female *A*. *eharai* were compared with literature values of other phytoseiids at 95% confidence level (Table 6). The attack rate of male *A*. *eharai* on *T*. *urticae* eggs was significantly lower than other predatory mites. The attack rate of female *A*. *eharai* on *T*. *urticae* eggs was not significantly different from that of *A*. *swirskii* (at 25°C) or *N*. *californicus* (at 25 and 30°C). The handling time of the predatory mites species included here on *T*. *urticae* eggs was the shortest for *N*. *longispinosus* at 35°C (0.264) and for female *A*. *eharai* (0.181). Female *A*. *eharai* showed a higher maximum attack rate (*T*/*T*_h_) on *T*. *urticae* eggs (132.74) than other phytoseiids, even higher than *N*. *longispinosus* at 35°C (90.91).

**Table 6:** Comparison of attack rates and handling times among the phytoseiid mites (*T* = 24h).

Prey stage	Species	Sex	Attack rate (*α* ± S.E.) (95% C.I.)	Handling time (*T*_h_ ± S.E.) (95% C.I.)	Maximum attack rate (*T*/*T*_h_)	Experimental conditions			Reference
						Temp. (°C)	Plant leaf (diameter, mm)	Mite age (days)	
Egg	*A*. *eharai*	F	0.055 ± 0.010de* (0.034–0.076)	0.181 ± 0.047g (0.086–0.276)	132.74	26.6	Kidney bean (35)	3	This study
		M	0.027 ± 0.009e (0.009–0.045)	0.578 ± 0.170defg (0.235–0.921)	41.53				
	*A*. *swirskii*	F	0.054 ± 0.003d (0.046–0.062)	0.706 ± 0.026e (0.654–0.758)	34.28	25.0	Cucumber (Unknown)	Unknown	[23]
	*N*. *californicus*	F	0.064 ± 0.004d (0.056–0.072)	1.655 ± 0.030a (1.596–1.713)	14.50	25.0	Strawberry (30)	5 to 7	[18]
			0.071 ± 0.008d (0.056–0.087)	1.331 ± 0.039b (1.253–1.409)	18.03	30.0			
			0.209 ± 0.032abc (0.145–0.273)	0.951 ± 0.021d (0.909–0.993)	25.23	35.0			
	*N*. *longispinosus*^†^	F	0.228 ± 0.018a (0.194–0.263)	0.768 ± 0.024e (0.744–0.816)	31.25	25.0	Bean (33)	7 to 10	[22]
			0.151 ± 0.011c (0.129–0.173)	0.576 ± 0.024f (0.528–0.600)	41.67	30.0			
			0.156 ± 0.007c (0.142–0.170)	0.264 ± 0.010g (0.264–0.288)	90.91	35.0			
	*N*. *womersleyi*^†^	F	0.228 ± 0.021ab (0.186–0.269)	1.344 ± 0.024b (1.272–1.392)	17.86	25.0			
			0.296 ± 0.027a (0.242–0.349)	1.200 ± 0.024c (1.152–1.248)	20.00	30.0			
			0.249 ± 0.018a (0.213–0.285)	1.248 ± 0.024bc (1.200–1.296)	19.23	35.0			
	*P*. *persimilis*	F	0.156 ± 0.017bc (0.123–0.190)	0.601 ± 0.015f (0.571–0.631)	39.95	25.0	Cucumber (35)	10 (from egg)	[21]
Larva	*A*. *eharai*	F	0.109 ± 0.023a (0.062–0.155)	0.164 ± 0.031c (0.102–0.226)	146.16	26.6	Kidney bean (35)	3	This study
		M	0.019 ± 0.004b (0.011–0.026)	0.234 ± 0.123c (-0.012–0.481)	102.43				
	*N*. *californicus*	F	0.068 ± 0.008a (0.052–0.083)	1.586 ± 0.053a (1.481–1.690)	15.14	25.0	Strawberry (30)	5 to 7	[18]
	*N*. *longispinosus*	F	0.026 ± 0.004b (0.018–0.033)	0.828 ± 0.087b (0.656–1.000)	28.98	25.0	Bean (35)	2	[19]

*Means followed by the same letter within a column in each prey stage are not significantly different at approximate 95% confidence interval.

^†^In the original literature, the time scale was 1 day, which converted to 24 hours in this study.

For larval *T*. *urticae* as prey, female *A*. *eharai* showed the highest attack rate (0.109) and the shortest handling time (0.164) among the compared phytoseiid mites. The attack rate of male *A*. *eharai* (0.019) was significantly lower than that of female *A*. *eharai*; however, there was no significance in handling time between the two sexes of this species. The maximum attack rates of both sexes of *A*. *eharai* (Female, 146.16; Male, 102.43) were higher than those of other phytoseiid mites considered.

Comparing *A*. *eharai* to other phytoseiids studied previously, *A*. *eharai* seems to have a higher predation capacity on *T*. *urticae* than *A*. *swirskii*, *N*. *californicus*, *N*. *womersleyi*, or *P*. *persimilis*, under similar conditions. Also, it has been suggested that the predation capacity of *A*. *eharai* might increase with adult age [20, 21]. Overall, *A*. *eharai* seems to have good potential for commercial development as a new natural enemy for augmentative biological control of *T*. *urticae* in greenhouses in mid-latitude region. In the studies we compared, there were variations in experimental layout such as plant species, size of leaf disc, mite age, and temperature. The test temperature for the referenced studies on *T*. *urticae* eggs or larvae was 25°C for all species of phytoseiids, and additionally 30 and 35°C were also tested for *N*. *californicus*, *N*. *longispinosus*, and *N*. *womersleyi* on *T*. *urticae* eggs as a prey (Table 6). However, the test temperature for *A*. *eharai* was 26.6°C in this study. The temperature would be one of the significant factors affecting the predation ability of predatory mites, as shown by the results for *N*. *californicus*, *N*. *longispinosus*, and *N*. *womersleyi* [18, 22]. These species showed higher predation efficiency at high temperatures. Nevertheless, *A*. *eharai* showed higher predation ability on *T*. *urticae*.

For *T*. *urticae* eggs, even though the attack rate of *A*. *eharai* was lowered compared to other predatory mites we considered, the handling time of adult females of *A*. *eharai* was significantly shorter than for *A*. *swirskii* (at 25°C), *N*. *californicus* (at 25, 30, and 35°C), *N*. *longispinosus* (at 25 and 30°C), *N*. *womersleyi* (at 25, 30, and 35°C), *P*. *persimilis* (at 25°C), and *N*. *californicus* (at 20 and 25°C). Also, in adult males of *A*. *eharai*, handling time for eggs as prey was shorter than for *N*. *womersleyi* (at 25, 30, and 35°C) and *P*. *persimilis* (at 25°C) (Table 6). To some degree these shorter handling times may compensate for the lower attack rates of *A*. *eharai*. Thus, the maximum attack rate (*T*/*T*_h_) of female *A*. *eharai* for *T*. *urticae* eggs was the highest among the compared phytoseiid mites, even higher than that of *N*. *californicus*, *N*. *longispinosus*, and *N*. *womersleyi* at 30 or 35°C.

Morphological structure on plant leaves such as density of trichomes can be a cause of variation in predation capacity of predatory mites [42], with some exception such as study by Ahn et al. [18] who found no difference in functional response of *N*. *californicus* against *T*. *urticae* between two varieties of strawberry with different leaf trichome densities. Also, age of phytoseiids can affect predation. Fathipour et al. [20] reported that the predatory capability of *A*. *swirskii* on *T*. *urticae* egg increased until 17 days after egg hatch of the predator at 25°C. Such a trend was also reported in *P*. *persimilis* [21]. The predation capacity of *P*. *persimilis* on *T*. *urticae* egg increased until this phytoseiid was 30 days old (post egg hatch) at 25°C. The phytoseiid predator age in our test was three days old after molting to adult (equivalent to 8 to 9 days old after egg hatch). Further research might be needed to determine whether the maximum predation capacity, as affected by maturation of the *A*. *eharai* individuals, was reached more quickly than by the other phytoseiids or if the predatory capacity of *A*. *eharai* was simply always higher even from an early age.

A temperature-dependent intrinsic rate of increase (*r*) model was constructed to predict the adaptability of *A*. *eharai* to temperature changes using the data of Park and Lee [11]. Also, to compare with *A*. *eharai*, the *r* models of other phytoseiid mites were extracted from referenced studies or constructed in this study using the data in references. For *A*. *eharai* and *N*. *californicus*, the *r* values at each temperature were fitted against temperatures using the Briere 1 model [43] to construct temperature-dependent *r* models by the program TableCurve 2D (SYSTAT Software Inc., San Jose, CA) [44]. And, for *A*. *swirskii*, *N*. *womersleyi*, and *N*. *longispinosus*, we used models from the original studies. The lower and upper thresholds and optimal temperatures for the population growth of all species were estimated and compared using the models. There was an error in the original research paper for the model of *N*. *longispinosus* [15]. Thus, it was reconstructed in this study using the same equation (Lactin 1) [45].

The lower- and upper-threshold, and optimal temperatures of selected phytoseiid mites from the literature were calculated using the temperature-dependent *r* model (Table 7). Among the compared mites, the optimal temperature for *r* was the lowest in *A*. *eharai* (28.1°C) followed by *N*. *californicus* (29.0°C), *A*. *swirskii* (30.1°C), *N*. *womersleyi* (30.5°C), and *N*. *longispinosus* (32.0°C). Also, *A*. *eharai* had the lowest upper-threshold temperature (33.2°C) and the narrowest temperature range (12.5 to 33.2°C) for population growth.

**Table 7:** The parameters and equations of temperature-dependent *r* models and lower- and upper threshold, and optimal temperatures for the two-spotted spider mite, *Tetranychus urticae*, and selected phytoseiid mites from this study and the literature.

Species	Prey	Temperature (°C)			Equation (*T* = temperature)	References for	
		Lower threshold	Optimal	Upper threshold		Model	Data
*A*. *eharai*	*T*. *urticae*	12.5	28.1	33.2	Briere 1: 0.00023*T* (*T*–12.47050) (33.18000–*T*)^1/2^	This study (*F*_2,3_ = 24.4, *P* = 0.014)	[11]
*A*. *swirskii*	Pollen	15.5	30.1	37.0	Briere 2: 0.0000943*T* (*T*–15.486) (36.992–*T*)^1/1.425^	[13]	[13]
*N*. *californicus*	*Panonychus ulmi*	7.6	29.0	35.2	Briere 1: 0.00019*T* (*T*–7.58920) (35.15485–*T*)^1/2^	This study (*F*_2,2_ = 111.4, *P* = 0.009)	[46]
*N*. *longispinosus*	*T*. *urticae*	13.3*	32.0	38.2	Lactin 1: *e*^0.162*T*^–*e*^0.162 × 38.2–(38.2–*T*) / 6.1517^ Linear: 0.0264*T*–0.3497	^†^This study (*F*_2,7_ = 53.40, *P* < 0.001)	[15]
*N*. *womersleyi*	*T*. *urticae*	12.5*	30.5	38.9	Lactin 2: *e*^0.023*T*^–*e*^0.023 × 44.9–(44.9–*T*) / 6.3843^–1.354 Linear: 0.0218*T*–0.272	[15]	[15]

*Calculated from the linear model.

^†^Re-constructed in this study using TableCurve 2D (SYSTAT Software Inc., San Jose, CA) [44] because of error in original study.

Many studies have examined temperature-dependent models of the developmental rate in immature stages (and for total fecundity) of various insects and mites [11, 43, 45, 47–49]. These models can be used to predict and better understand the population dynamics of insects and mites. However, the mites have a very short life cycle; therefore, an *r* value that included information for both the immature and adult stages would be a better parameter to use in constructing a temperature-dependent model for mites. In comparing temperature-dependent *r* models among phytoseiids, *A*. *eharai* showed a slightly narrower temperature range for development (12.5 to 33.2°C) than did the other species considered (Table 7). Moreover, the optimal temperature of *A*. *eharai* (28.1°C, Table 7) was the lowest among the other mites we compared. The functional response studies on *N*. *californicus*, *N*. *longispinosus*, and *N*. *womersleyi* to *T*. *urticae* eggs were conducted at three temperatures (25, 30, and 35°C) (Table 6). *Neoseiulus californicus* showed a higher predation rate at 35°C, and this rate did not correspond with the optimal temperature (29.0°C) for *N*. *californicus* in the temperature-dependent *r* model (Tables 6 and 7). However, the higher predation rate of *N*. *womersleyi* (30°C) and *N*. *longispinosus* (35°C) seemed to match their optimal temperatures, 30.5 and 32.0°C, respectively (Tables 6 and 7). The optimal temperature for population development by *A*. *eharai* was 28.1°C. This means that this predator can reach faster to its maximum performance at the season of low temperature than the other phytoseiids because they had a lower optimal temperature. Generally, the mean temperature in greenhouses in mid-latitude region does not exceed 30°C, even in the summer season [50]. Thus, *A*. *eharai* would have a high potential as a biological control agent in the greenhouses.

## Conclusion

In conclusion, *A*. *eharai*, a native species in Far East Asia and the dominant phytoseiid in crops in Korea [7, 16], has a high potential as a new biological control agent because of its higher predation capacity. Moreover, as this predatory mite has a relatively lower optimal temperature for population growth, it would confer an advantage for the use at the season of low temperature. Lastly, further studies on additional topics, such as this predator’s response to other pests or an outdoor field study, or a greenhouse crop study, are needed to advance our understanding of this species’ potential for commercial use.

## Supporting information

S1 Data (XLSX)
